# Association of Cystatin C- and Creatinine-Based Estimated Glomerular Filtration Rate With Adverse Outcomes in Heart Failure With Preserved Ejection Fraction

**DOI:** 10.1016/j.xkme.2026.101435

**Published:** 2026-06-15

**Authors:** Bethany Roehm, Xiaoyue Zhang, Jie Yang, John Haley, Justin L. Grodin, S. Susan Hedayati

**Affiliations:** 1 Division of Nephrology, University of Texas Southwestern Medical Center, Dallas, TX; 2 Biostatistical Consulting Core, Stony Brook University, Renaissance School of Medicine, Stony Brook, NY; 3 Department of Family, Population, and Preventative Medicine, Stony Brook University, Renaissance School of Medicine, Stony Brook, NY; 4 Department of Pathology, Stony Brook University, Renaissance School of Medicine, Stony Brook, NY; 5 Division of Cardiology, University of Texas Southwestern Medical Center, Dallas, TX; 6 Division of Nephrology and Hypertension, Stony Brook University, Renaissance School of Medicine, Stony Brook, NY

**Keywords:** eGFR, cystatin C, cardiovascular outcomes, HFpEF, heart failure

## Abstract

**Rationale and Objective:**

Cystatin C-based versus creatinine-based measures of estimated glomerular filtration rate (eGFR) are more predictive of worse outcomes in people with heart failure with reduced ejection fraction (HFrEF). It is unknown if cystatin C-based measures may also yield more accurate prognoses in heart failure with preserved ejection fraction (HFpEF).

**Study Design:**

A longitudinal analysis of people with HFpEF.

**Setting & Participants:**

214 participants from the Treatment of Preserved Cardiac Function Heart Failure with an Aldosterone Antagonist trial with available serum at baseline and 12 months in the National Heart, Lung, and Blood Institute’s Biologic Specimen and Data Repository Information Coordinating Center.

**Exposures:**

Baseline, time-varying, or change over 12 months in eGFRcr, eGFRcys, and eGFRcr-cys

**Outcomes:**

Composite of aborted cardiac arrest, heart failure hospitalization, or cardiovascular death.

**Analytical Approach:**

Fine-Gray and joint models.

**Results:**

Median follow-up was (IQR) 3.09 (2.07-4.44) years. All 3 baseline eGFR measures were independently associated with the risk of the composite endpoint, after adjustment for race, diabetes mellitus, and body mass index: HR (95% CI) per 5mL/min/1.73m^2^ lower eGFR: eGFRcr, 1.16 (1.04-1.28); eGFRcys, 1.20 (1.08-1.34); eGFRcr-cys, 1.19 (1.08-1.32). After adjusting for NT-proBNP and age, no baseline eGFR measure was statistically significantly associated with the composite endpoint. Only a relative decline in eGFRcr-cys from baseline to 12 months was associated with the composite endpoint in fully adjusted models: eGFRcr-cys, HR 1.31 (1.02-1.67); per 10% eGFR decrease). Lower eGFR at any timepoint, using all 3 measures, was associated with the composite endpoint in fully-adjusted models.

**Limitations:**

small number of events, possible survival bias

**Conclusions:**

A decline in eGFRcr-cys from baseline to 12 months is associated with adverse cardiovascular outcomes in people wit reduced ejection fraction h HFrEF. Measures of eGFR that incorporate only cystatin C may not be of greater prognostic value in people with HFpEF than measures that also incorporate serum creatinine.

## Plain-Language Summary

Cystatin C-based measures of kidney function have been more predictive of adverse outcomes in people with heart failure with reduced ejection fraction (HFrEF), but their prognostic utility in heart failure with preserved ejection fraction (HFpEF) is unclear. In a longitudinal analysis of 214 participants from the Treatment of Preserved Cardiac Function Heart Failure with an Aldosterone Antagonist trial, a HFpEF population, we found that a relative decline in eGFRcr-cys from baseline to 12 months was associated with adverse cardiovascular outcomes. However, cystatin C-based measures on their own were not associated with adverse cardiovascular outcomes. This suggests that cystatin C based measures may not be more prognostic that serum creatinine-based measures in HFpEF.

Lower estimated glomerular filtration rate (eGFR) using serum creatinine (Scr) is associated with a greater risk of mortality and HF hospitalizations in people with heart failure (HF) with preserved ejection fraction (HFpEF).^1^ More recent data have shown that eGFR measures derived using cystatin C (eGFRcr-cys or eGFRcys) may provide better estimates of kidney function in populations with heart failure with reduced ejection fraction (HFrEF).^2^^,^^3^ Additionally, we and others showed that discordance between eGFRcr and eGFRcys (defined as an eGFRcys that is at least 30% lower than eGFRcr)^4^ is associated with adverse outcomes among people with HFpEF hospitalized with acute HF and those with HFrEF.^5^^,^^6^ However, it is unknown if cystatin C-based measures of eGFR on their own may be more strongly associated with adverse outcomes in an ambulatory HFpEF population.

The rationale for enhanced risk prediction of cystatin C-based eGFR measures in HF has been attributed to the influence of muscle mass, which may be reduced in HF patients with sarcopenia, onScr. These patients may be less capable of generating creatinine independent of kidney function, yielding a higher eGFRcr. Although sarcopenia is associated with greater mortality in both HFrEF and HFpEF, it is less-well established whether people with HFpEF are as much affected with sarcopenia as those with HFrEF, with meta-analyses yielding conflicting results.^7^^,^^8^ Moreover, most studies of eGFR in HFpEF utilized eGFRcr only when determining associations of kidney function with adverse outcomes.

The aim of this study was to evaluate whether baseline eGFR including eGFR discordance, time-varying eGFR, or the change from baseline in eGFR, as measured by eGFRcr, eGFRcys, or eGFRcr-cys, was associated with a composite of aborted cardiac arrest, HF hospitalization, or cardiovascular (CV) death, in a HFpEF population. We hypothesized that lower baseline eGFR or a decline in eGFR from baseline to 12 months would be associated with a higher risk of a composite CV outcome, regardless of whether eGFR measure.

## Methods

### Study Design and Population

**Figure 1:**
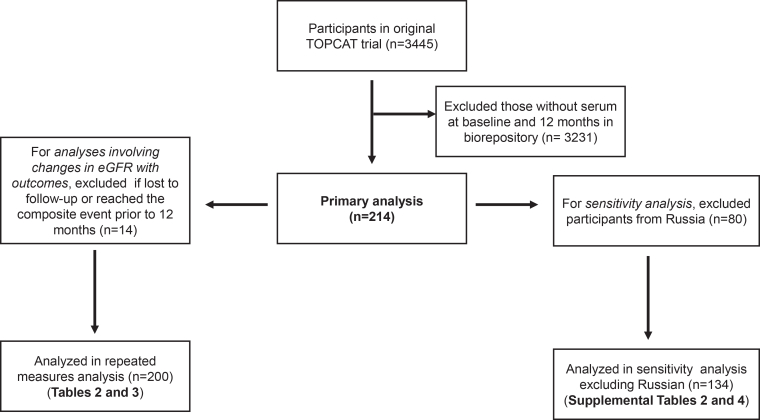
Study eligibility flow diagram. A flow diagram is shown to depict how participants from the original TOPCAT study were selected for this analysis.

We analyzed data and performed biomarker analyses on participants with HFpEF from the Treatment of Preserved Cardiac Function Heart Failure with an Aldosterone Antagonist trial (TOPCAT). Details of this study have been previously published.^9^ Per the original manuscript for the TOPCAT study, all enrolled participants provided written informed consent, and the ethics committee from each study site approved the trial design. For this analysis, this was deemed by the local IRB to be exempt research since it utilized de-identified data. Samples were obtained from the National Heart, Lung, and Blood Institute’s Biologic Specimen and Data Repository Information Coordinating Center (BioLINCC). We included all participants who had serum samples available at both baseline and 12 months (Fig 1). We did not exclude participants for any other reason in initial analyses.

Briefly, TOPCAT was a randomized, double-blind controlled trial whereby 3,445 participants age 50 or older with HFpEF were randomized to receive spironolactone vs. placebo to evaluate whether spironolactone had any effect on a prespecified composite outcome of CV death, aborted cardiac arrest, or HF hospitalization. Participants had a left ventricular ejection fraction ≥45 % and either a history of HF hospitalization in the preceding 12 months or elevated natriuretic peptides within 60 days prior to randomization.^9^ Patients were ineligible if eGFRcr was <30 mL/min/1.73m^2^, Scr ≥2.5 mg/dL, or serum potassium was ≥5.0 mmol/L. All participants provided written informed consent. Oversight was provided by an ethics committee at each study site. Participants in our analysis were from patients enrolled from the United States, Canada, and Russia and had similar characteristics as compared with the rest of the sample from these 3 countries (N = 2,329, results not shown).

### Serum Creatinine and Cystatin C Assessment

Scr was previously measured as described in the TOPCAT Manual of Operations. We used frozen serum samples to measure cystatin C. Cystatin C assays were performed using the turbidimetric method at the University of Minnesota Advanced Research and Diagnostic Laboratory, Minneapolis, MN, using the Gentian cystatin C Reagent Kit REF 1101 (Gentian ASA) on the Roche Cobas 8000 Chemistry analyzer (Roche Diagnostics Corporation). eGFRcr and eGFRcr-cys were calculated using the 2021 CKD EPI equations, and eGFRcys was calculated using the 2012 CKD EPI equation.^2^^,^^10^

### N-terminal pro-B-Type Natriuretic Peptide Assessment

The Olink Proximity Extension Assay PCR-based protein analysis instrument was to measure N-terminal pro-B-type natriuretic peptide (NT-proBNP) from frozen serum. One microliter of serum was incubated overnight with antibody pairs labelled with DNA oligonucleotides. Oligonucleotides based paired by proximity on the protein target were extended using thermostable DNA polymerase and amplified. The pre-amplified target was measured by qPCR in a 384-well microfluidics chip. Internal, incubation, and detection controls were used to validate analysis integrity. A pooled serum sample was used as an additional control. Data analysis was performed using Olink NPX Signature algorithms (v1.13), allowing quality control and internal standard normalization. Protein expression was expressed in arbitrary log2 units (Normalized Protein eXpression - NPX), derived from the qPCR Ct values.

### Outcome Measures

The prespecified primary outcome for this study was a composite of CV death, aborted cardiac arrest, or HF hospitalization, as in the original TOPCAT study.

### Statistical Analysis

Descriptive analyses were conducted with categorical data described as counts and proportions, and continuous data presented as median (25th, 75th percentile). Participants were stratified by whether they reached the composite event during follow-up. Chi-square tests with exact *P*-values based on Monte-Carlo simulation were utilized to compare categorical variables across different groups. For continuous variables, Wilcoxon rank sum tests were utilized.

Cumulative incidence curves were used to describe time to composite outcome with death due to other causes being a competing risk event. Baseline eGFRcr, eGFRcys, and eGFRcr-cys were categorized into 3 groups (<45, 45-59, ≥60 mL/min/1.73m^2^). The *cuminc() f*unction in the R *cmprsk* package was used to estimate the cumulative incidences of the composite outcome within different patient groups. The comparison of cumulative incidence curves was performed using Gray’s test. Unadjusted and adjusted proportional sub-distribution hazards models (Fine-Gray models) were utilized to examine the association between baseline eGFR with the risk of the composite outcome. eGFRcr, eGFRcys, and eGFRcr-cys were considered as continuous variables in regression models. Discordant eGFR was treated dichotomously with eGFRcys/eGFRcr <0.7 indicative of eGFR discordance.^6^ Covariates associated with the composite outcome based on univariate analyses, including race, diabetes, and NT-proBNP, were included in the adjusted models. BMI was also included as a proxy for obesity since obesity is associated with cystatin C.^11^ Treatment group was adjusted in the final multivariable regression model. As an exploratory analysis, an interaction term between eGFR and treatment was added to the regression models to evaluate for effect modification. Sensitivity analysis was done including age as a covariate. Another sensitivity analysis excluded Russian samples, given the heterogeneity of TOPCAT results observed in people recruited from Russia.^12^

Fine-Gray models were constructed to evaluate the association between changes in eGFR at month 12 from baseline and the composite outcome. Absolute and relative changes in eGFR were analyzed. Absolute change was defined as the difference between eGFR values at month 12 and baseline (Month 12-Baseline). Relative change was calculated as the absolute change divided by the baseline value [Month 12-Baseline]/Baseline). These analyses included only the subgroup of participants who survived through month 12 and did not experience the composite event prior to month 12. The models were adjusted for the same covariates used previously, further including the baseline eGFR values.

Joint models were used to assess the association between time-varying eGFR and the composite outcome. The longitudinal sub-model consisted of a linear mixed-effects model with both random intercept and random slope for time, capturing eGFR measurements at baseline and at month 12. The survival sub-model employed a Fine-Gray model to evaluate the association of both the current value and slope of eGFR with the composite outcome. No covariates were included in the longitudinal sub-model. The survival sub-model adjusted for race, diabetes, BMI, NT-proBNP (NPX), and treatment group. The *jm()* function in the *JMbayes2* R package was used with adaptive Gauss-Hermite quadrature to estimate the joint model parameters. Marginal Watanabe-Akaike Information Criterion compared model fit, with lower values indicating better fit.

For all Fine-Gray models, C-indices were calculated using the linear predictors from the models, representing the proportion of concordant pairs among possible pairs of individuals, including those that are concordant, discordant, and tied for the event of interest. C-indices of models incorporating different eGFR measures but adjusted for the same set of covariates were compared using bootstrapping with 5,000 resamples with replacement. Each *P*-value was determined as the proportion of simulations where the difference in c-indices from the simulated datasets was greater than the difference observed using the original data.

To handle a potential violation of the proportional hazards assumption, as implied by the cumulative incidence curves of composite outcome across 3 levels of baseline eGFRcys, exploratory analyses were performed assessing Schoenfeld residuals for the continuous eGFRcys variable from Fine-Gray models with adjustment of an interaction term between time cutoff variable (at 0.5, 1, 1.5, 2, or 2.5 years) and baseline eGFRcys. More stable Schoenfeld residuals with values closer to zero indicated that the proportional hazards assumption was not violated. Based on the Akaike Information Criterion (AIC), the model using a one-year cutoff provided the best fit to the data. Sensitivity analyses were performed exploring the association of eGFRcys with the composite outcome, including a time cutoff at 12 months and its interaction with eGFRcys in the adjusted models.

Statistical analyses were performed using SAS 9.4 (SAS Institute Inc) Significance level was set at 0.05.

## Results

The baseline characteristics are shown in Table 1 stratified by whether participants experienced the composite outcome. Participants were followed for a median (IQR) of 3.09 (2.07-4.44) years of whom 35 (16.4%) experienced a composite event. Twenty-seven participants (12.6%) experienced a HF hospitalization, of whom 8 subsequently died from CV causes. Eight participants died from CV causes without prior HF hospitalization. One participant experienced aborted cardiac arrest, which occurred following HF hospitalization. Median age (interquartile range, IQR) was 72 (62-78) years, median eGFRcr-cys 61 (47-73) mL/min/1.73 m^2^, 48% of participants had eGFRcr-cys <60, and 21% had eGFRcr-cys <45 mL/min/1.73 m^2^. Thirty-three percent had diabetes mellitus, 37.4% had New York Heart Association Functional Classification (NYHA) III/IV HF, and 81.3% were receiving renin-angiotensin-aldosterone system inhibitors.

**Table 1:** Baseline characteristics stratified by composite outcome

Characteristic	Total (N = 214)	No Composite Outcome (n = 179)	Composite Outcome^a^ (n = 35)	*P*^b^
**Spironolactone treatment Group**	100 (46.7%)	82 (45.8%)	18 (51.4%)	0.54
**Age (y)**	72 (62-78)	71 (62-78)	74 (62-80)	0.15
**Female**	103 (48.1%)	82 (45.8%)	21 (60.0%)	0.12
**Race**				
Black	15 (7.0%)	6 (3.4%)	9 (25.7%)	< 0.0001
Other	199 (93.0%)	173 (96.6%)	26 (74.3%)	
**NYHA class**				
I & II	134 (62.6%)	115 (64.2%)	19 (54.3%)	0.27
III & IV	80 (37.4%)	64 (35.8%)	16 (45.7%)	
**BMI (kg/m^2^)**	31.5 (27.4-35.6)	31.2 (27.1-34.5)	32.9 (27.4-38.7)	0.10
**Hypertension**	200 (93.5%)	167 (93.3%)	33 (94.3%)	1.00
**Diabetes**	71 (33.2%)	50 (27.9%)	21 (60.0%)	0.0002
**Smoking**	17 (7.9%)	15 (8.4%)	2 (5.7%)	0.75
**RAASi**	174 (81.3%)	148 (82.7%)	26 (74.3%)	0.24
**β-Blocker**	180 (84.1%)	149 (83.2%)	31 (88.6%)	0.47
**Diuretic use**	177 (82.7%)	143 (79.9%)	34 (97.1%)	0.01
**NT-proBNP (NPX)**	5.1 (4.0-6.0)	4.8 (3.8-5.9)	6.0 (4.7-7.2)	0.0003
**Creatinine (mg/dL)**	1.1 (0.9-1.2)	1.1 (0.9-1.2)	1.2 (1.0-1.6)	0.003
**Cystatin C (mg/L)**	1.3 (1.1-1.6)	1.2 (1.1-1.5)	1.5 (1.2-2.4)	0.0002
**eGFRcr (mL/min/1.73m^2^)**	64.6 (53.6-77.8)	66.1 (55.6-80.0)	54.5 (39.2-69.6)	0.0008
**eGFRcys (mL/min/1.73m^2^) m^2^)**	52.2 (39.1-66.4)	55.2 (40.7-67.7)	40.2 (22.9-55.1)	0.0001
**eGFRcr-cys (mL/min/1.73m^2^) m^2^)**	61.1 (47.3-73.2)	63.4 (49.2-74.7)	50.3 (29.1-63.8)	< 0.0001
**eGFRcr (mL/min/1.73m^2^)**				
<45	28 (13.1%)	17 (9.5%)	11 (31.4%)	0.0009
45-59	55 (25.7%)	45 (25.1%)	10 (28.6%)	
≥60	131 (61.2%)	117 (65.4%)	14 (40.0%)	
**eGFRcys (mL/min/1.73m^2^)**				
<45	81 (37.9%)	59 (33.0%)	22 (62.9%)	0.003
45-59	57 (26.6%)	50 (27.9%)	7 (20.0%)	
≥60	76 (35.5%)	70 (39.1%)	6 (17.1%)	
**eGFRcr-cys (mL/min/1.73m^2^)**				
<45	45 (21.0%)	29 (16.2%)	16 (45.7%)	0.0003
45-59	58 (27.1%)	49 (27.4%)	9 (25.7%)	
≥60	111 (51.9%)	101 (56.4%)	10 (28.6%)	
**UACR**^c^**(mg/g)**	19.5 (8.8-75.1)	18.0 (8.8-70.7)	26.5 (9.0-79.6)	0.49

Abbreviations: BMI, body mass index; eGFRcr, creatinine-based estimated glomerular filtration rate; eGFRcys, cystatin C-based estimated glomerular filtration rate; eGFRcr-cys, creatinine and cystatin C-based estimated glomerular filtration rate; NYHA, New York Heart Association; NT-proBNP, N-terminal pro-B-type natriuretic peptide; NPX, normalized protein expression; RAASi, renin-angiotensin-aldosterone system inhibitors; UACR, urine albumin-to-creatinine ratio.

a Outcome was a composite of cardiovascular death, aborted cardiac arrest, or heart failure hospitalization.

b For categorical variables, p-values were from χ^2^ tests with Monte-Carlo simulation; for continuous variables, *P* values were from Wilcoxon rank-sum tests.

c UACR was missing in 126 (58.9%) participants. 14 (11.1%) of participants with a missing UACR had a composite outcome. Column percentages were reported for categorical variables, median (Q1, Q3) were reported for continuous variables.

**Figure 2:**
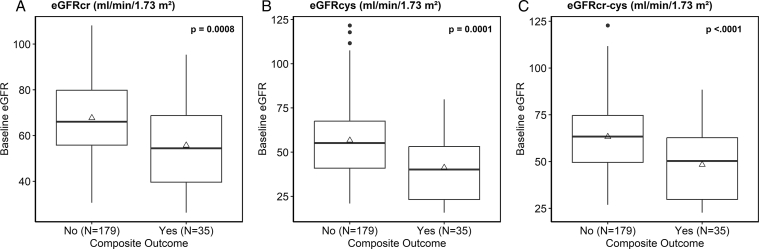
Baseline eGFR Measures Stratified by Composite Outcome. Boxplots are shown for baseline eGFRcr (panel A), eGFRcys (panel B), and eGFRcr-cys (panel C). Each eGFR measures is stratified by those who did versus did not experience the composite outcome of cardiovascular death, aborted cardiac arrest, or heart failure hospitalization.

The baseline eGFR using all 3 measures was lower (Fig 2) and NT-proBNP was higher among participants who experienced the composite outcome vs. those who did not. A higher proportion of those who experienced the composite outcome were Black, had diabetes mellitus, and were prescribed diuretics versus those who did not. There were no statistically significant differences in age, sex, BMI, or albuminuria.

Baseline characteristics among those who experienced the composite event after 12 months are shown in Table 2. Eleven participants experienced the composite outcome prior to 12 months, and 3 were lost to follow-up prior to 12 months (Fig 1). Participants who experienced an event were older, had a higher proportion who were Black or had diabetes mellitus, and had a higher NT-proBNP level vs. those who did not. The median (IQR) change from baseline to 12 months in eGFRcr was −8.5 (−13.2 to 2.9) mL/min/1.73m^2^ among those with an event vs. −3.3 (−11.4 to 5.3) mL/min/1.73m^2^ among those did not, although not statistically significance (Table 2). Median (IQR) change in eGFRcys was −5.3 (−10.3 to −0.7) mL/min/1.73m^2^ among those with an event versus −3.3 (−9.3 to 2.4) mL/min/1.73m^2^ among those who did not, also not significant.

**Table 2:** Characteristics of Participants who Experienced a Composite Outcome After 12 Months

Variable	Total (N = 200)	No (n = 176)	Had Composite Event^a^ (N=24)	*P*^b^
**Spironolactone treatment group**	92 (46.0%)	81 (46.0%)	11 (45.8%)	0.99
**Age (y)**	72 (62-78)	71 (62-77)	78 (65.5-82.5)	0.02
**Female**	93 (46.5%)	81 (46.0%)	12 (50.0%)	0.71
**Race**				
Black	10 (5.0%)	6 (3.4%)	4 (16.7%)	0.02
Other	190 (95.0%)	170 (96.6%)	20 (83.3%)	
**NYHA class**				
I & II	126 (63.0%)	113 (64.2%)	13 (54.2%)	0.34
III & IV	74 (37.0%)	63 (35.8%)	11 (45.8%)	
**BMI (kg/m^2^)**	31.3 (27.4-35.4)	31.2 (27.3-34.6)	32.0 (28.4-38.1)	0.25
**Hypertension**	186 (93.0%)	164 (93.2%)	22 (91.7%)	1
**Diabetes**	63 (31.5%)	49 (27.8%)	14 (58.3%)	0.003
**Smoking**	16 (8.0%)	15 (8.5%)	1 (4.2%)	0.7
**RAASi**	164 (82.0%)	145 (82.4%)	19 (79.2%)	0.7
**β-Blocker**	167 (83.5%)	146 (83.0%)	21 (87.5%)	0.77
**Diuretic use**	163 (81.5%)	140 (79.5%)	23 (95.8%)	0.09
**NT-proBNP (NPX)**	5.0 (3.9-6.0)	4.8 (3.8-5.9)	6.2 (4.9-7.1)	0.0007
**Creatinine (mg/dL)**	1.1 (0.9-1.2)	1.1 (0.9-1.2)	1.2 (1.0-1.5)	0.01
**Cystatin C**^c^**(mg/L)**	1.2 (1.1-1.6)	1.2 (1.1-1.5)	1.5 (1.2-2.3)	0.001
**Baseline eGFRcr (mL/min/1.73m^2^)**	65.0 (54.2-78.1)	66.1 (55.5-79.8)	58.2 (41.0-68.7)	0.01
**Baseline eGFRcr (mL/min/1.73m^2^)**				0.01
<45	24 (12.0%)	17 (9.7%)	7 (29.2%)	
45-59	50 (25.0%)	44 (25.0%)	6 (25.0%)	
≥60	126 (63.0%)	115 (65.3%)	11 (45.8%)	
**Month-12 eGFRcr (mL/min/1.73m^2^)**	62.3 (49.0-76.0)	63.5 (50.6-78.4)	49.4 (41.4-60.8)	0.0006
**Absolute change in eGFRcr (mL/min/1.73m^2^)**	−3.5 (−12.0 to 4.6)	−3.3 (−11.4 to 5.3)	−8.5 (−13.2 to 2.9)	0.27
**Relative change in eGFRcr (%)**	−4.2 (−18.7 to 8.2)	−4.1 (−15.3 to 9.3)	−10.1 (−24.1 to −0.6)	0.12
**n (%) with decrease in eGFRcr from baseline to 12 months**	130 (65.0%)	111 (63.1%)	19 (79.2%)	0.12
**Baseline eGFRcys (mL/min/1.73 m^2^)**	53.6 (39.5-67.1)	55.2 (40.9-68.1)	41.2 (23.2-56.1)	0.0009
**Baseline eGFRcys (mL/min/1.73 m^2^)**				0.01
<45	72 (36.0%)	57 (32.4%)	15 (62.5%)	
45-59	54 (27.0%)	49 (27.8%)	5 (20.8%)	
≥60	74 (37.0%)	70 (39.8%)	4 (16.7%)	
**Month-12 eGFRcys (mL/min/1.73m^2^)**	49.6 (35.4-63.7)	53.0 (38.6-65.6)	35.3 (21.8-46.1)	<0.0001
**Absolute change in eGFRcys (mL/min/1.73m^2^)**	−3.5 (−9.6 to 2.1)	−3.3 (−9.3 to 2.4)	−5.3 (−10.3 to −0.7)	0.26
**Relative change in eGFRcys (%)**	−6.0 (−20.4 to 4.2)	−5.3 (−19.9 to 5.2)	−13.1 (−24.3 to −2.2)	0.07
**n (%) with decrease in eGFRcys from baseline to 12 months**	130 (65.7%)	111 (63.8%)	19 (79.2%)	0.17
**Baseline eGFRcr-cys (mL/min/1.73m^2^)**	61.5 (47.6-73.4)	63.5 (49.1-74.9)	50.5 (31.1-64.8)	0.001
**Baseline eGFRcr-cys (mL/min/1.73m^2^)**				0.01
<45	39 (19.5%)	29 (16.5%)	10 (41.7%)	0.01
45-59	53 (26.5%)	47 (26.7%)	6 (25.0%)	
≥60	108 (54.0%)	100 (56.8%)	8 (33.3%)	
**Month-12 eGFRcr-cys (mL/min/1.73m^2^)**	57.9 (43.6-72.1)	61.9 (45.4-73.7)	45.9 (29.7-53.4)	<0.0001
**Absolute change in eGFRcr-cys (mL/min/1.73m^2^)**	−2.8 (−10.0 to 2.6)	−2.3 (−9.5 to 3.4)	−4.3 (−13.1 to 1.1)	0.17
**Relative change in eGFRcr-cys (%)**	−4.2 (−17.0 to 4.6)	−3.6 (−15.7to 6.2)	−11.4 (−23.5 to 2.4)	0.05
**n (%) decrease in eGFRcr-cys from baseline to 12 months**	129 (65.2%)	112 (64.4%)	17 (70.8%)	0.65
**UACR**^d^**(mg/g)**	17.8 (8.8-79.6)	17.8 (8.8-69.9)	16.0 (8.4-167.5)	1

Abbreviations: BMI, body mass index; eGFRcr, creatinine-based estimated glomerular filtration rate; eGFRcys, cystatin C-based estimated glomerular filtration rate; eGFRcr-cys, creatinine and cystatin C-based estimated glomerular filtration rate; NYHA, New York Heart Association; NT-proBNP, N-terminal pro-B-type natriuretic peptide; NPX, normalized protein expression; RAASi, renin-angiotensin-aldosterone system inhibitors; UACR, urine albumin-to-creatinine ratio.

a Outcome was a composite of cardiovascular death, aborted cardiac arrest, or heart failure hospitalization.

b For categorical variables, *P*-values were from χ^2^ tests with Monte-Carlo simulation; for continuous variables, *P*-values were from Wilcoxon rank-sum tests.

c There were 2 participants who did not have Cystatin C values available at month-12. As result, their eGFRcys and eGFRcr-cys values were also missing at that time point, along with the corresponding absolute changes, relative changes and decrease indicators. Both participants were in the group without a composite outcome.

d UACR was missing in 120 (60%) participants at baseline. 8 (6.7%) of the participants with a missing UACR had a composite outcome. Column percentages were reported for categorical variables, median (Q1, Q3) were reported for continuous variables.

### Association of Baseline eGFR with the Composite Outcome

**Figure 3:**
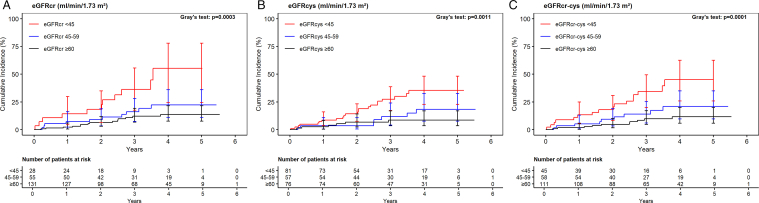
Cumulative incidence of experience the composite event stratified by baseline eGFR. Cumulative incidence curves showing the probability of experiencing the composite outcome of cardiovascular death, aborted cardiac arrest, or heart failure hospitalization over the follow-up period, accounting for competing risk of all-cause death, for each eGFR measure – eGFRcr (Panel A), eGFRcys (Panel B), eGFRcr-cys (Panel C) – are shown. Each eGFR measure is further stratified by eGFR <45, 45-59, or ≥60 mL/min/1.73m^2^.

Cumulative incidence survival curves demonstrated a greater risk of the composite outcome among those with lower baseline eGFRcr, eGFRcys, or eGFRcr-cys (Fig 3). Point estimates for the cumulative incidence of experiencing the composite event stratified by year are shown in Table S1. Lower baseline values of all three eGFR measures were associated with a greater risk of the composite outcome in unadjusted analyses using Fine-Gray models (Table 3). These associations remained statistically significant in adjusted analyses controlling for race, diabetes mellitus, and BMI. However, after adjusting for NT-proBNP and treatment group, only eGFRcr-cys remained statistically significantly associated with the composite outcome (HR 1.15; 95% CI, 1.00-1.31; *P* = 0.04, per 5 mL/min/1.73m^2^ lower baseline eGFRcr-cys). In a sensitivity analysis adjusting for age, none of the baseline eGFR measures were associated with the composite outcome (Table S2). Discordant eGFR was not associated with the composite on adjusted analysis (HR 1.52; 95% CI, 0.64-3.59; *P* = 0.34). Subgroup analysis using non-Russian samples suggested the same direction and similar magnitude of associations between the three baseline eGFR measures and the composite endpoint (eGFRcr: HR 1.12; 95% CI, 0.99-1.29; *P* = 0.07; eGFRcys: HR 1.15; 95% CI, 0.98-1.35; *P* = 0.09; eGFRcr-cys: HR 1.15; 95% CI, 0.99-1.34; *P* = 0.07, per 5 mL/min/1.73m^2^ lower baseline eGFR) (Table S3). There was no statistically significant difference in model C-indices between any of the eGFR measures (Table S4). There was a violation in the proportional hazard assumption across 3 levels of eGFRcys. Further exploration revealed that the risk of the composite event was significantly higher after 12 months among those with a lower baseline eGFRcys, even in fully adjusted models, but not during the first year (Table 4). There was no interaction by treatment (data not shown).

**Table 3:** Association of eGFR With the Composite Outcome

eGFR	HR (95% CI)	*P*	C-Index	HR (95% CI)	*P*	C-Index	HR (95% CI)	*P*	C-Index	HR (95% CI)	*P*	C-Index	HR (95% CI)	*P*	C-Index
Baseline eGFR^a^															
	Unadjusted			Model I^b^			Model II^c^			Model III^d^			Model IV^e^		
eGFRcr	1.20 (1.08-1.34)	0.001	0.66	1.16 (1.04-1.28)	0.005	0.7	1.16 (1.04-1.28)	0.005	0.71	1.12 (0.99-1.26)	0.07	0.77	1.12 (0.99-1.27)	0.07	0.77
eGFRcys	1.25 (1.11-1.41)	0.0003	0.68	1.20 (1.08-1.34)	0.001	0.74	1.20 (1.08-1.34)	0.001	0.75	1.15 (1.00-1.31)	0.05	0.78	1.15 (1.00-1.33)	0.05	0.78
eGFRcr-cys	1.25 (1.12-1.40)	< 0.0001	0.69	1.20 (1.08-1.33)	0.001	0.74	1.19 (1.08-1.32)	0.0007	0.74	1.15 (1.00-1.31)	0.04	0.78	1.15 (1.00-1.33)	0.047	0.77
eGFRcys/eGFRcr <0.7	2.70 (1.23-5.93)	0.01	0.6	2.53 (1.11-5.76)	0.03	0.6	2.52 (1.11-5.74)	0.03	0.59	1.47 (0.63-3.44)	0.37	0.74	1.52 (0.64-3.59)	0.34	0.75

Absolute Change in eGFR^f^															
	Model 0^g^			Model I^h^			Model II^i^			Model III^j^			Model IV^k^		
eGFRcr	1.24 (1.07-1.43)	0.005	0.68	1.19 (1.04-1.37)	0.01	0.68	1.20 (1.03-1.41)	0.02	0.67	1.21 (1.02-1.44)	0.03	0.75	1.21 (1.03-1.43)	0.02	0.75
eGFRcys	1.30 (1.05-1.61)	0.02	0.72	1.24 (1.01-1.51)	0.04	0.71	1.25 (1.01-1.55)	0.04	0.71	1.23 (0.98-1.55)	0.08	0.75	1.23 (0.98-1.55)	0.08	0.75
eGFRcr-cys	1.32 (1.08-1.61)	0.006	0.72	1.26 (1.04-1.53)	0.02	0.71	1.28 (1.03-1.58)	0.02	0.71	1.27 (1.02-1.59)	0.03	0.76	1.28 (1.02-1.60)	0.03	0.76

Relative Change in eGFR^l^															
	Model 0^g^			Model I^h^			Model II^i^			Model III^j^			Model IV^k^		
eGFRcr	1.29 (1.10-1.51)	0.001	0.69	1.21 (1.04-1.42)	0.02	0.67	1.24 (1.03-1.50)	0.02	0.66	1.27 (1.03-1.56)	0.02	0.75	1.28 (1.05-1.57)	0.02	0.76
eGFRcys	1.25 (1.05-1.50)	0.01	0.71	1.20 (1.01-1.42)	0.04	0.71	1.22 (1.00-1.49)	0.04	0.71	1.22 (1.00-1.50)	0.05	0.75	1.22 (1.00-1.51)	0.06	0.75
eGFRcr-cys	1.32 (1.10-1.59)	0.003	0.71	1.25 (1.04-1.50)	0.02	0.71	1.29 (1.04-1.61)	0.02	0.71	1.31 (1.05-1.64)	0.02	0.76	1.32 (1.05-1.65)	0.02	0.77

Abbreviations: BMI, body mass index; eGFR, estimated glomerular filtration rate; eGFRcr, creatinine-based estimated glomerular filtration rate; eGFRcys, cystatin C-based estimated glomerular filtration rate; eGFRcr-cys, creatinine and cystatin C-based estimated glomerular filtration rate; NT-proBNP, N-terminal pro-B-type natriuretic peptide.

a For baseline eGFR, reported HRs, and 95% CI corresponded to every 5 mL/min/1.73m^2^ lower baseline values.

b Model I, adjusted for race and diabetes.

c Model II: Model I + BMI.

d Model III: Model II + NT-proBNP.

e Model IV: Model III + treatment group.

f For absolute change in eGFR at 12 months, 3 participants were not included because they were lost to follow-up prior to 12 months; 11 were not included because they reached the composite event prior to 12 months. Reported HRs and 95% CI corresponded to every 5 mL/min/1.73m^2^ decrease from baseline to month-12.

g Model 0, adjusted for baseline GFR.

h Model I: Model 0 + race and diabetes.

i Model II: Model I + BMI.

j Model III: Model II + NT-proBNP.

k Model IV: Model III + treatment group.

l For relative change in eGFR at 12 months, 3 participants were not included because they were lost to follow-up prior to 12 months; 11 were not included because they reached the composite event prior to 12 months. Reported HRs and 95% CI corresponded to every 10% decrease from baseline to month-12.

**Table 4:** Association of Baseline eGFRcys With the Composite Outcome With Adjustment of an Interaction Term Between eGFRcys and Time ≥1 vs <1 Year

Model	Time Cutoff	HR (95% CI) for Composite Outcome	*P*	*P*-value for Interaction Term Between eGFRcys and Time	C-Index
Base^a^	<1 y	1.02 (0.85-1.22)	0.84	0.06	0.81
	≥1 y	1.27 (1.10-1.46)	0.001		
Model I^b^	<1 y	0.98 (0.85-1.14)	0.83	0.03	0.82
	≥1 y	1.23 (1.08-1.40)	0.002		
Model II^c^	<1 y	0.98 (0.85-1.14)	0.84	0.02	0.82
	≥1 y	1.23 (1.08-1.40)	0.002		
Model III^d^	<1 y	1.02 (0.85-1.23)	0.82	0.28	0.84
	≥1 y	1.18 (1.02-1.38)	0.03		
Model IV^e^	<1 y	1.04 (0.84-1.29)	0.73	0.4	0.85
	≥1 y	1.18 (1.01-1.37)	0.04		

*Note:* Two hundred and fourteen participants were included. Reported HRs and 95% CI corresponded to every 5 mL/min/1.73m^2^ decrease in baseline eGFRcys value.

a Adjusted for time cutoff at 1 year and an interaction term between eGFRcys and the time cutoff.

b Model I: Base + race and diabetes.

c Model II: Model I + BMI.

d Model III: Model II + NT-proBNP.

e Model IV: Model III + treatment group.

### Association of Change in eGFR with Composite Outcome

An absolute decrease in eGFR from baseline to month 12 was associated with an increased risk of the composite outcome for all 3 eGFR measures, after adjusting for baseline eGFR, race, diabetes mellitus, and BMI (Table 3). However, after further adjustment for NT-proBNP and treatment group, only eGFRcr and eGFRcr-cys remained statistically significantly associated with the composite outcome (eGFRcr: HR 1.21; 95% CI, 1.03-1.43; *P* = 0.02; eGFRcr-cys: HR 1.28; 95% CI, 1.02-1.60; *P* = 0.03; per 5 mL/min/1.73m^2^). No eGFR measure was associated with the composite outcome on sensitivity analysis including age as a covariate. In fully adjusted models including NT-proBNP and treatment group, only a 10% relative decrease in eGFRcr and eGFRcr-cys were statistically significantly associated with the composite outcome (eGFRcr: HR 1.28; 95% CI, 1.05-1.57; *P* = 0.02; eGFRcr-cys: HR 1.32; 95% CI, 1.05-1.65; *P* = 0.02) (Table 3). These associations were not modified by treatment (data not shown). In a sensitivity analysis adjusting for age, only eGFRcr-cys was associated with the composite outcome (HR 1.31; 95% CI, 1.02-1.67; *P* = 0.03) (Table S2). Subgroup analysis using non-Russian samples suggested the same direction and similar magnitude of associations between the change of eGFR measures and the composite endpoint (Table S3). There was no statistically significant difference in C-index between any of the eGFR measures (Table S4).

### Association of Time-varying eGFR with Composite Outcome

Joint model analysis was done to account for changing eGFR over time. From models without including the slope of eGFR in survival sub-models, both unadjusted and adjusted analyses—considering race, diabetes, NT-proBNP, and treatment arm—showed that lower eGFR at any time point, on all 3 eGFR measures, was associated with the composite outcome (Table 5) (eGFRcr: HR 1.20; 95% CI, 1.06-1.39; *P* = 0.002; eGFRcys: HR 1.23; 95% CI, 1.07-1.44; *P* = 0.002; eGFRcr-cys: HR 1.22; 95% CI, 1.08-1.44; *P* = 0.001; per every 5 mL/min/1.73 m^2^ decrease in eGFR). However, at any time during the observation period, the rate of change in eGFR (slope) did not provide more information about the risk of the composite CV events (Table 5). Results were similar in sensitivity analysis using non-Russian samples (Table S5).

**Table 5:** Joint Models for the Association Between Time-Varying eGFR and Composite Outcome

eGFR	Variable	Unadjusted			Model I^a^			Model II^b^			Model III^c^			Model IV^d^		
		HR (95% CI)	*P*	Marginal WAIC	HR (95% CI)	*P*	Marginal WAIC	HR (95% CI)	*P*	Marginal WAIC	HR (95% CI)	*P*	Marginal WAIC	HR (95% CI)	*P*	Marginal WAIC
**Model with current value of eGFR**																
eGFRcr	Current value	1.29 (1.12-1.52)	< 0.0001	2,541.6	1.21 (1.08-1.39)	0.0002	2,517.2	1.22 (1.08-1.39)	0.0003	3,278.1	1.19 (1.06-1.37)	0.003	2,521.9	1.20 (1.06-1.39)	0.002	2,614.9
eGFRcys	Current value	1.33 (1.16-1.56)	< 0.0001	2,518.5	1.28 (1.12-1.47)	< 0.0001	2,590.2	1.28 (1.12-1.48)	< 0.0001	2,507.1	1.22 (1.07-1.42)	0.002	3,038.4	1.23 (1.07-1.44)	0.002	2,591.1
eGFRcr-cys	Current value	1.31 (1.15-1.52)	< 0.0001	2,418.9	1.26 (1.12-1.42)	< 0.0001	2,486.9	1.26 (1.12-1.43)	< 0.0001	2,557.5	1.21 (1.08-1.38)	0.0009	2,430.1	1.22 (1.08-1.41)	0.001	2,509.6
**Model with both current value and slope of eGFR**																
eGFRcr	Current value	1.28 (1.05-1.57)	0.02	2,535.3	1.23 (1.01-1.47)	0.04	3,067.5	1.23 (1.03-1.51)	0.03	2,518	1.17 (0.89-1.60)	0.25	6,959,403,050.7	1.18 (0.92-1.61)	0.19	33,78559.3
	Slope	1.25 (0.30-6.05)	0.75		1.18 (0.22-10.08)	0.95		1.12 (0.20-8.39)	0.96		3.31 (0.04-38.70)	0.33		2.30 (0.06-30.81)	0.49	
eGFRcys	Current value	1.35 (1.15-1.61)	0.0008	2,677.4	1.29 (1.10-1.55)	0.01	1,543,610.8	1.30 (1.10-1.58)	0.01	3,068.7	1.25 (0.91-1.78)	0.12	42,417,151.6	1.26 (0.95-1.65)	0.08	5,706,610.5
	Slope	1.17 (0.20-9.35)	0.90		0.82 (0.06-11.25)	0.74		0.80 (0.04-17.14)	0.75		0.67 (0.00-126.00)	0.94		1.05 (0.01-72.84)	0.87	
eGFRcr-cys	Current value	1.31 (1.08-1.56)	0.02	2,541.1	1.27 (1.10-1.52)	0.003	19,356	1.27 (1.08-1.54)	0.008	2,455.7	1.22 (0.94-1.72)	0.12	20,404,777	1.26 (0.97-1.76)	0.08	1,738,564
	Slope	1.56 (0.25-18.00)	0.64		0.91 (0.06-15.33)	0.85		1.21 (0.07-22.21)	0.90		8.18 (0.01-899.97)	0.34		1.56 (0.01-245.35)	0.76	

*Note:* Two hundred and fourteen participants were included. HRs and 95% CI of the current GFR value corresponded to every 5 mL/min/1.73m^2^ decrease in eGFR; HRs and 95% CI of the slope corresponded to every 5 mL/min/1.73m^2^/year decrease in the changing rate.

Abbreviation: WAIC, Watanabe–Akaike information criterion.

a Model I: adjusted for race and diabetes.

b Model II: Model I + baseline BMI.

c Model III: Model II + NT-proBNP.

d Model IV: Model III + treatment group.

## Discussion

The key observation from this study was that in ambulatory patients with HFpEF a relative decrease over 12 months in eGFR, only as measured by eGFRcr-cys, was associated with adverse CV outcomes in fully adjusted models. Of the 3 baseline eGFR measures—eGFRcr, eGFRcr-cys, and eGFRcys—only eGFRcr-cys, was associated with a greater risk of adverse CV events in adjusted models that incorporated NT-proBNP. However, this association was attenuated in a sensitivity analysis adjusting for age. These findings suggest that measures of eGFR that incorporate only cystatin C may not be of better prognostic value in people with HFpEF than measures that also incorporateScr.

Plausible explanations exist regarding why eGFR measures that only incorporated cystatin C and eGFR discordance did not perform as well in HFpEF as in other populations for prognosticating CV outcomes.^3^^,^^6^^,^^13^, ^14^, ^15^ First, cystatin C is less influenced by muscle mass than Scr.^16^ eGFRcr may be higher than true GFR in patients with sarcopenia, such as in HFrEF, resulting in a comparatively lower eGFRcys versus a patient without sarcopenia. However, interpreting the contribution of sarcopenia to Scr in HFpEF is challenging. Data comparing the prevalence of sarcopenia in HFpEF versus HFrEF is equivocal. Some studies have shown a higher prevalence of sarcopenia in HFrEF versus HFpEF, though others have not.^7^^,^^8^^,^^17^ Additionally, sarcopenia patterns may differ between people with HFpEF versus HFrEF.^8^ For example, low appendicular skeletal muscle mass may be more prevalent in those with HFrEF, while people with HFpEF are more likely to have low handgrip strength and gait speed.^7^ Given these complexities, studies incorporating measured GFR and measures of sarcopenia in HFpEF are needed.

Moreover, eGFRcys may not be an accurate estimate of kidney function in people with HFpEF with obesity. Obesity is common in HFpEF, affecting 55%-80%; metabolic syndrome affects >50% of people with HFpEF.^18^^,^^19^ Adipocytes release cystatin C at higher levels than non-adipose tissue.^20^ Obesity and metabolic syndrome are both associated with higher cystatin C levels independent of kidney function.^11^^,^^21^ Additionally, inflammation has been associated with higher cystatin C levels independent of kidney function.^22^^,^^23^ Obesity and metabolic syndrome are both proinflammatory conditions.^24^ In our analysis, obesity was common, with a median BMI of 31.5 kg/m^2^. It is, therefore, plausible that eGFRcys may be lower in some individuals due to the contribution of obesity to endogenous cystatin C levels rather than a true decrement in kidney function, thus affecting its prognostic utility.

Overall, we found a relative decline in eGFRcr-cys over 12 months was associated with adverse outcomes in HFpEF. Given that sarcopenia may lead to higher eGFRcr measurements and obesity to lower eGFRcys measurements, the weighted average of the two may yield a better estimate of kidney function in HFpEF. In support of this, a study of >6,000 adults, including people with CV disease and HF referred for measured GFR found among those with discordant eGFRcr and eGFRcys, and eGFRcr-cys most closely associated with measured GFR.^25^ Additionally, the 2024 KDIGO guidelines recommend use of eGFRcr-cys for evaluation of the association of eGFR with clinical outcomes.^26^

In our analysis, though a relative decrease in eGFRcr-cys was associated with the composite outcome, eGFR slope was not. Other studies have found an association between either decline in eGFR or eGFR slope with a greater risk of adverse outcomes in HF. Supporting the findings in our study, an analysis using electronic health records data of 1946 patients with HF, 39% of whom had HFpEF, showed every 10 mL/min/1.73m^2^ decrease in eGFR was associated with a 22% increase in mortality over a median of 3.16 years.^27^ An analysis of the Atherosclerosis Risk in Communities study found that, over a median of 6 years, steeper eGFR slope was associated with greater risk of incident HF events among a high risk population.^28^ We may not have been able to find an association between eGFR slope and the composite outcome for a few reasons. First, the low event rate may have limited our power. Second, we only had two time points available to measure eGFR slope, baseline and 12 months, which may reflect regression to the mean or be too soon to detect a significant longitudinal decline in eGFR. Third, TOPCAT excluded people with a baseline eGFRcr <30 mL/min/1.73m^2^, a population more likely to experience a steeper slope.

Our study has several strengths. We studied eGFR measures that incorporate both Scrand cystatin C in an HFpEF population. Additionally, we were able to evaluate these measures at multiple time points. Finally, we were able to adjust for NT-proBNP, for which there has historically been a significant amount of missingness in TOPCAT. However, we independently ran NT-proBNP assays so had values for all participants in our sample. Adjusting for NT-proBNP significantly affected our results since NT-proBNP is a strong predictor of adverse outcomes and treatment response in HFpEF.^29^, ^30^, ^31^, ^32^ Limitations include a small number of events, which did not allow for adjustment for multiple comparisons and may have limited our ability to detect an association with the composite outcome. Therefore, these results need to be validated in larger cohorts. Inclusion only of participants with samples available at baseline and 12 months for cystatin C may have introduced survival bias. Finally, post hoc analysis of TOPCAT revealed geographic heterogeneity of spironolactone adherence and inclusion criteria by natriuretic peptide levels in Russia.^12^ Our sensitivity analyses excluding Russia revealed similar results.

In conclusion, a decline in eGFRcr-cys from baseline to 12 months was associated with adverse CV outcomes in HFpEF. However, eGFRcys and discordance in eGFR were not. Although published data support the use of cystatin C-based eGFR measures in HFrEF,^33^ the use of eGFRcys on its own in HFpEF needs validation in larger cohorts and compared with measured GFR due to the potential effects of obesity on cystatin C levels in this population.
