# Corrigendum to “Association of Cystatin C- and Creatinine-Based eGFR With Adverse Outcomes in Heart Failure With Preserved Ejection Fraction” Kidney Medicine (2026) Article 101435; available online 15 June 2026

**DOI:** 10.1016/j.xkme.2026.101483

**Published:** 2026-07-29

**Authors:** Bethany Roehm, Xiaoyue Zhang, Jie Yang, John Haley, Justin L. Grodin, S. Susan Hedayati

**Affiliations:** 1 Division of Nephrology, University of Texas Southwestern Medical Center, Dallas, TX; 2 Biostatistical Consulting Core, Stony Brook University, Renaissance School of Medicine, Stony Brook, NY; 3 Department of Family, Population, and Preventative Medicine, Stony Brook University, Renaissance School of Medicine, Stony Brook, NY; 4 Department of Pathology, Stony Brook University, Renaissance School of Medicine, Stony Brook, NY; 5 Division of Cardiology, University of Texas Southwestern Medical Center, Dallas, TX; 6 Division of Nephrology and Hypertension, Stony Brook University, Renaissance School of Medicine, Stony Brook, NY

The authors regret that the first sentence of the Conclusions section in the Abstract incorrectly stated: “A decline in eGFRcr-cys from baseline to 12 months is associated with adverse cardiovascular outcomes in people with HFrEF.” The sentence should read: “A decline in eGFRcr-cys from baseline to 12 months is associated with adverse cardiovascular outcomes in people with HFpEF.”

The authors would like to apologize for any inconvenience caused.

DOI of original article: 10.1016/j.xkme.2026.101435

