# Supplementary material for: Association of Cystatin C- and Creatinine-Based Estimated Glomerular Filtration Rate With Adverse Outcomes in Heart Failure With Preserved Ejection Fraction
Source: Kidney Med. 2026 Jun 15;8(8):101435. doi: 10.1016/j.xkme.2026.101435 (PMC13396861; doi:10.1016/j.xkme.2026.101435)
Supplement: Supplementary File (PDF) — Tables S1-S5 [file mmc1.pdf]

Supplementary Material

Table S1. Estimated unadjusted cumulative incidence with 95% CI of composite outcome at year 1 through 5 after randomization stratified by eGFR categories

		**Year**			
**eGFR**	**Level**	**1**	**2**	**3**	**4**
eGFRcr (ml/min/1.73 m^2)	< 45	14.3% (4.4%, 29.8%)	18.4% (6.5%, 35.0%)	36.4% (17.5%, 55.6%)	55.3% (24.5%, 77.9%)
	45-59	7.3% (2.3%, 16.2%)	9.2% (3.4%, 18.8%)	16.2% (7.4%, 28.0%)	22.4% (11.1%, 36.1%)
	≥ 60	2.3% (0.6%, 6.0%)	6.5% (3.0%, 11.8%)	12.1% (6.7%, 19.3%)	13.7% (7.7%, 21.5%)
eGFRcys (ml/min/1.73 m^2)	< 45	8.6% (3.8%, 16.0%)	14.3% (7.5%, 23.2%)	27.5% (17.3%, 38.6%)	35.4% (22.8%, 48.2%)
	45-59	3.5% (0.6%, 10.8%)	3.5% (0.6%, 10.8%)	11.8% (4.1%, 24.0%)	18.4% (7.7%, 32.7%)
	≥ 60	2.6% (0.5%, 8.3%)	6.8% (2.5%, 14.2%)	8.7% (3.5%, 16.9%)	8.7% (3.5%, 16.9%)
eGFRcr-cys (ml/min/1.73 m^2)	< 45	13.3% (5.3%, 25.0%)	18.0% (8.3%, 30.7%)	34.5% (20.0%, 49.5%)	45.1% (25.9%, 62.5%)
	45-59	5.2% (1.3%, 13.1%)	9.3% (3.4%, 19.0%)	14.0% (6.0%, 25.3%)	20.9% (9.7%, 35.0%)
	≥ 60	1.8% (0.3%, 5.8%)	4.7% (1.7%, 9.9%)	9.8% (4.7%, 17.1%)	11.7% (5.8%, 19.8%)
Note: No participants experienced the composite outcome after year 4. Therefore, the estimated cumulative incidence at year 5 is identical to that at year 4 and is not shown separately.					

Table S2. Association of eGFR with the composite outcome with adjustment of age

	Baseline eGFR[1]			Absolute Change in eGFR[2]			Relative Change in eGFR[3]		
**eGFR**	**HR (95% CI)**	**P-value**	**C-index**	**HR (95% CI)**	**P-value**	**C-index**	**HR (95% CI)**	**P-value**	**C-index**
eGFRcr	1.09 (0.95 - 1.26)	0.21	0.77	1.19 (0.98 - 1.44)	0.08	0.76	1.24 (0.99 - 1.55)	0.06	0.76
eGFRcys	1.11 (0.95 - 1.31)	0.18	0.78	1.22 (0.94 - 1.58)	0.13	0.76	1.22 (0.97 - 1.53)	0.08	0.77
eGFRcr-cys	1.12 (0.96 - 1.31)	0.16	0.78	1.26 (0.99 - 1.62)	0.06	0.77	1.31 (1.02 - 1.67)	0.03	0.77

[1] For baseline eGFR, reported HRs and 95% CI corresponded to every 5 ml/min/1.73 m^2 lower baseline values. Model adjusted for race, diabetes, BMI, NT-proBNP, treatment group, and age at entering the study.

[2] For absolute change in eGFR at 12 months, 3 participants were not included because they were lost to follow-up prior to 12 months; 11 were not included because they reached the composite event prior to 12 months. Reported HRs and 95% CI corresponded to every 5 mL/min/1.73 m^2 decrease from baseline to month-12. Model adjusted for baseline GFR, race, diabetes, BMI, NT-proBNP, treatment group, and age at entering the study.

[3] For relative change in eGFR at 12 months, 3 participants were not included because they were lost to follow-up prior to 12 months; 11 were not included because they reached the composite event prior to 12 months. Reported HRs and 95% CI corresponded to every 10% decrease from baseline to month-12. Model adjusted for baseline GFR, race, diabetes, BMI, NT-proBNP, treatment group, and age at entering the study.

eGFR, estimated glomerular filtration rate; eGFRcr, creatinine-based estimated glomerular filtration rate; *eGFRcys*, cystatin C-based estimated glomerular filtration rate; *eGFRcr-cys,* creatinine and cystatin C-based estimated glomerular filtration rate; *BMI*, body mass index; *NT-proBNP*, N-terminal pro-B-type natriuretic peptide.

Table S3. Association of eGFR with the composite outcome among non-Russian participants

eGFR	HR (95% CI)	P-value	C-index	HR (95% CI)	P-value	C-index	HR (95% CI)	P-value	C-index	HR (95% CI)	P-value	C-index	HR (95% CI)	P-value	C-index
**Baseline eGFR[1] (N = 134)**															
	**Unadjusted**			**Model I[2]**			**Model II[3]**			**Model III[4]**			**Model IV[5]**		
eGFRcr	1.16 (1.05 - 1.29)	0.005	0.64	1.14 (1.03 - 1.26)	0.009	0.68	1.14 (1.03 - 1.27)	0.01	0.70	1.12 (0.99 - 1.26)	0.06	0.74	1.12 (0.99 - 1.28)	0.07	0.74
eGFRcys	1.20 (1.05 - 1.38)	0.008	0.65	1.19 (1.05 - 1.34)	0.006	0.71	1.18 (1.05 - 1.33)	0.007	0.72	1.15 (0.98 - 1.33)	0.08	0.75	1.15 (0.98 - 1.35)	0.09	0.75
eGFRcr-cys	1.20 (1.06 - 1.35)	0.003	0.67	1.18 (1.05 - 1.32)	0.004	0.71	1.17 (1.05 - 1.31)	0.005	0.72	1.14 (0.99 - 1.31)	0.06	0.75	1.15 (0.99 - 1.34)	0.07	0.75
**Absolute Change in eGFR[6] (N =120)**															
	**Model 0[7]**			**Model I[8]**			**Model II[9]**			**Model III[10]**			**Model IV[11]**		
eGFRcr	1.10 (0.93 - 1.30)	0.27	0.64	1.06 (0.91 - 1.24)	0.4187	0.62	1.07 (0.90 - 1.28)	0.42	0.64	1.09 (0.91 - 1.31)	0.35	0.70	1.09 (0.91 - 1.31)	0.32	0.70
eGFRcys	1.30 (0.94 - 1.80)	0.11	0.69	1.23 (0.91 - 1.67)	0.1844	0.69	1.27 (0.90 - 1.78)	0.17	0.70	1.29 (0.91 - 1.83)	0.16	0.73	1.30 (0.90 - 1.87)	0.16	0.75
eGFRcr-cys	1.20 (0.92 - 1.56)	0.17	0.68	1.14 (0.89 - 1.47)	0.2992	0.68	1.17 (0.87 - 1.57)	0.29	0.68	1.20 (0.89 - 1.61)	0.23	0.72	1.21 (0.89 - 1.63)	0.22	0.72
**Relative Change in eGFR[12] (N =120)**															
	**Model 0[7]**			**Model I[8]**			**Model II[9]**			**Model III[10]**			**Model IV[11]**		
eGFRcr	1.15 (0.97 - 1.35)	0.10	0.64	1.10 (0.95 - 1.27)	0.21	0.63	1.13 (0.93 - 1.35)	0.21	0.65	1.16 (0.94 - 1.42)	0.17	0.70	1.16 (0.95 - 1.42)	0.14	0.71
eGFRcys	1.24 (0.98 - 1.57)	0.07	0.70	1.18 (0.95 - 1.48)	0.13	0.70	1.24 (0.94 - 1.65)	0.13	0.70	1.28 (0.96 - 1.70)	0.09	0.74	1.29 (0.95 - 1.75)	0.10	0.75

eGFR	HR (95% CI)	P-value	C-index	HR (95% CI)	P-value	C-index	HR (95% CI)	P-value	C-index	HR (95% CI)	P-value	C-index	HR (95% CI)	P-value	C-index
eGFRcr-cys	1.22 (0.99 - 1.51)	0.06	0.69	1.16 (0.96 - 1.41)	0.13	0.68	1.22 (0.94 - 1.59)	0.13	0.68	1.27 (0.97 - 1.66)	0.08	0.72	1.28 (0.97 - 1.71)	0.08	0.73

[1] For baseline eGFR, reported HRs and 95% CI corresponded to every 5 ml/min/1.73 m^2 lower baseline values.
[2] Model I: adjusted for race and diabetes.
[3] Model II: Model I + BMI
[4] Model III: Model II + NT-proBNP.
[5] Model IV: Model III + treatment group.
[6] For absolute change in eGFR, reported HRs and 95% CI corresponded to every 5 mL/min/1.73 m^2 decrease from baseline to month-12.
[7] Model 0, adjusted for baseline GFR.
[8] Model I: Model 0 + race and diabetes.
[9] Model II: Model I + BMI.
[10] Model III: Model II + NT-proBNP.
[11] Model IV: Model III + treatment group.
[12] For relative change in eGFR, reported HRs and 95% CI corresponded to every 10% decrease from baseline to month-12.

eGFR, estimated glomerular filtration rate; eGFRcr, creatinine-based estimated glomerular filtration rate; *eGFRcys*, cystatin C-based estimated glomerular filtration rate; *eGFRcr-cys,* creatinine and cystatin C-based estimated glomerular filtration rate; *BMI*, body mass index; *NT-proBNP*, N-terminal pro-B-type natriuretic peptide.

Table S4. P-values comparing C-indices of Fine-Gray models for association of eGFR with composite outcome

	P-values				
**Comparison of C-index shown in Table 2**	**Unadjusted**	**Model I[1]**	**Model II[2]**	**Model III[3]**	**Model IV[4]**
**Baseline eGFR**					
eGFRcr-cys vs. eGFRcr	0.48	0.48	0.46	0.45	0.64
eGFRcr-cys vs. eGFRcys	0.50	0.43	0.45	0.43	0.49
eGFRcys vs. eGFRcr	0.48	0.51	0.48	0.49	0.61
**Absolute Change in eGFR**					
	**Model 0[5]**	**Model I[6]**	**Model II[7]**	**Model III[8]**	**Model IV[9]**
eGFRcr-cys vs. eGFRcr	0.44	0.57	0.46	0.50	0.45
eGFRcr-cys vs. eGFRcys	0.50	0.49	0.48	0.39	0.43
eGFRcys vs. eGFRcr	0.46	0.56	0.48	0.56	0.50
**Relative Change in eGFR**					
	**Model 0[5]**	**Model I[6]**	**Model II[7]**	**Model III[8]**	**Model IV[9]**
eGFRcr-cys vs. eGFRcr	0.46	0.35	0.51	0.44	0.46
eGFRcr-cys vs. eGFRcys	0.47	0.47	0.36	0.23	0.47
eGFRcys vs. eGFRcr	0.48	0.40	0.59	0.64	0.48

[1] Model I: adjusted for race and diabetes.

[2] Model II: Model I + BMI.

[3] Model III: Model II + NT-proBNP.

[4] Model IV: Model III + treatment group.

[5] Model 0: adjusted for baseline GFR.

[6] Model I: Model 0 + race and diabetes.

[7] Model II: Model I + BMI.

[8] Model III: Model II + NT-proBNP.

[9] Model IV: Model III + treatment group.

P-values are calculated using bootstrapping (n=5000 resamples with replacement).

Table S5. Joint models for the association between time-varying eGFR and composite outcome among non-Russian participants

eGFR	Variable	Unadjusted			Model I[1]			Model II[2]			Model III[3]			Model IV[4]		
		HR (95% CI)	P-value	Marginal WAIC	HR (95% CI)	P-value	Marginal WAIC	HR (95% CI)	P-value	Marginal WAIC	HR (95% CI)	P-value	Marginal WAIC	HR (95% CI)	P-value	Marginal WAIC
Model with current value of eGFR																
eGFRcr	Current value	1.21 (1.04 - 1.41)	0.008	2662.5	1.17 (1.04 - 1.34)	0.004	2447	1.18 (1.05 - 1.34)	0.005	1876.6	1.16 (1.03 - 1.33)	0.02	2081	1.17 (1.03 - 1.35)	0.03	1949.5
eGFRcys	Current value	1.29 (1.09 - 1.55)	0.003	3595.8	1.25 (1.09 - 1.47)	0.0004	2416	1.26 (1.10 - 1.47)	0.0005	1861	1.21 (1.05 - 1.44)	0.01	1683.7	1.23 (1.06 - 1.47)	0.007	1705.2
eGFRcr-cys	Current value	1.26 (1.08 - 1.48)	0.002	1630	1.22 (1.08 - 1.40)	0.0006	3480	1.22 (1.08 - 1.40)	0.0009	1726.3	1.20 (1.05 - 1.39)	0.007	1780	1.21 (1.05 - 1.41)	0.006	1706.8
Model with both current value and slope of eGFR																
eGFRcr	Current value	1.23 (1.03 - 1.53)	0.03	1650.6	1.22 (1.03 - 1.55)	0.03	1811.4	1.23 (1.02 - 1.55)	0.04	5357.5	1.28 (1.00 - 1.70)	0.048	115158.9	1.31 (0.94 - 1.82)	0.09	390395.3
	Slope	0.80 (0.11 - 4.94)	0.72		0.64 (0.06 - 7.21)	0.54		0.52 (0.03 - 3.99)	0.47		0.20 (0.02 - 7.07)	0.32		0.09 (0.01 - 2.17)	0.12	
eGFRcys	Current value	1.29 (1.06 - 1.59)	0.02	3657.4	1.22 (0.79 - 1.56)	0.15	13936.8	1.23 (0.82 - 1.57)	0.14	51427.9	1.18 (0.71 - 1.76)	0.37	93167179.2	1.03 (0.50 - 1.65)	0.78	35560180.8
	Slope	0.99 (0.09 - 14.07)	0.91		0.35 (0.00 - 6.57)	0.53		0.41 (0.00 - 9.01)	0.57		0.25 (0.00 - 241.51)	0.71		0.02 (0.00 - 10.81)	0.16	
eGFRcr-cys	Current value	1.27 (1.06 - 1.54)	0.01	1663.4	1.26 (1.05 - 1.62)	0.02	2451.6	1.26 (0.98 - 1.64)	0.06	2755.8	1.28 (0.95 - 1.86)	0.10	13926107	1.25 (0.76 - 1.90)	0.24	3615612
	Slope	0.86 (0.07 - 8.59)	0.81		0.43 (0.01 - 19.58)	0.49		0.28 (0.00 - 6.61)	0.41		0.15 (0.00 - 93.05)	0.47		0.02 (0.00 - 5.30)	0.16	

[1] Model I: adjusted for race and diabetes.
[2] Model II: Model I + baseline BMI.
[3] Model III: Model II + NT-proBNP.
[4] Model IV: Model III + treatment group.
HRs and 95% CI of the current GFR value corresponded to every 5 mL/min/1.73 m² decrease in eGFR; HRs and 95% CI of the slope corresponded to every 5 mL/min/1.73 m²/year decrease in the changing rate.
WAIC, Watanabe–Akaike information criterion.
